# Potential role of *STAG1* mutations in genetic predisposition to childhood hematological malignancies

**DOI:** 10.1038/s41408-022-00683-9

**Published:** 2022-06-02

**Authors:** Claudia Saitta, Stefano Rebellato, Laura Rachele Bettini, Giovanni Giudici, Nicolò Panini, Eugenio Erba, Valentina Massa, Franziska Auer, Ulrike Friedrich, Julia Hauer, Andrea Biondi, Grazia Fazio, Giovanni Cazzaniga

**Affiliations:** 1 Centro Ricerca M. Tettamanti, Pediatrics, University of Milano Bicocca, Monza, Italy; 2 Department of Oncology, Istituto di Ricerche Farmacologiche Mario Negri, IRCCS Milan, Milan, Italy; 3 Department of Health Sciences, University of Milan, Milan, Italy; 4 Technical University of Munich, School of Medicine, Department of Pediatrics, München, Germany; 5 National Center for Tumor Diseases (NCT); Faculty of Medicine and University Hospital Carl Gustav Carus, Technische Universität Dresden, Dresden, Germany; 6 Pediatrics, University of Milano Bicocca, Fondazione MBBM/San Gerardo Hospital, Monza, Italy; 7 Medical Genetics, University of Milano Bicocca, School of Medicine and Surgery, Monza, Italy

**Keywords:** Cancer genetics, Oncogenesis

## Dear Editor,

Cohesin ring is a multi-protein complex that plays an essential role in a wide range of cellular processes: besides its canonical role in sister chromatids cohesion and segregation [1], the complex gives a fundamental contribution to DNA repair and maintenance of genome integrity [2], and in transcriptional regulation [3]. Cohesin genes are classified as encoding core subunits (*SMC1A*, *SMC3*, *RAD21*, and the paralogs *STAG1*/*STAG2)*, and cohesin regulatory factors (e.g., *NIPBL*, *HDAC8*, and others) [1, 2].

Among these, *STAG1* encodes for a key subunit of the complex, essential for chromatids cohesion [1, 4].

Germline mutations of cohesins lead to Cohesinopathies [5], while recurrent somatic mutations in multiple components of the complex are known in myelodysplastic syndromes (MDS) and acute myeloid leukemia (AML) [6], as well as solid tumors [7].

A correlation between Cohesinopaties and cancer predisposition has not been established yet. However, the reports of three Cornelia de Lange patients (CdLS) affected respectively by Down syndrome-like acute megakaryoblastic leukemia (AMKL) [8], acute lymphoblastic leukemia (ALL) [9] and myelodysplastic syndrome (MDS) [10], suggest that germline mutations in Cohesins could constitute a predisposing factor to hematological disorders.

The present study aims to characterize germline Cohesins variants in pediatric patients affected by hematological diseases.

We screened 120 childhood ALL consecutive diagnoses: 107 B-ALL (89.1%), 11 T-ALL (9.2%), and 2 mixed phenotype acute leukemia cases (MPAL) (1.7%). Additionally, we sequenced 19 sporadic pediatric patients referred by our clinicians for having a familial recurrence of cancer (*n* = 8), syndromic features (*n* = 9) associated with either ALL (*n* = 15) or AML (*n* = 2); two additional cases were rare pediatric MDS.

A custom Next-Generation Sequencing panel was used, including 39 genes associated with predisposition and leukemogenesis. (Supplementary Table 1). We sequenced DNA extracted from bone marrow mononuclear cells during the disease and remission phase, the latter defined by a minimal residual disease (MRD) value below 10^−4^. NGS data that support the findings of this study are available in the ArrayExpress database (www.ebi.ac.uk/arrayexpress), reference numbers E-MTAB-11757 and E-MTAB-11760.

We focused on Cohesins variants and two previously uncharacterized heterozygous variants have been identified in the *STAG1* gene.

The first *STAG1* variant (Arg1167Gln) was found in a 2 years old male patient affected by BII-ALL (negative for common translocations; central nervous system negative; medium risk for MRD). He was enrolled in the AIEOP-BFM ALL2009 protocol, he experienced a late combined relapse (BM and CNS) and underwent HSCT. The patient had no comorbidities nor syndromic stigmata.

The second variant (STAG1 Arg1187Gln) was identified in a 14 years old male patient affected by MDS with an excess of blasts (MDS-EB1), with complex karyotype (47, XY,+8, del(16)(q22)[19]/46, XY[1]), who received HSCT. The patient did not show any syndromic features.

Both *STAG1* variants are located in a highly conserved region of the gene, frequently affected by mutations known to be implicated in oncogenesis (Pecan database, https://pecan.stjude.cloud/STAG1) (Fig. S1A).

The Arg1167Gln (c.3500G > A; rs747617236) is a germline missense alteration (VAF 44.6% at diagnosis and 41.3% in remission), classified as VUS in InterVar and Varsome. It is the only variant identified, among genes included in the NGS panel.

The Arg1187Gln (c.3560G > A; rs777032446) is a germline missense variant (VAF 51.9% at diagnosis) predicted as likely pathogenic in Varsome and VUS in InterVar. We validated the germline origin by PCR and Sanger sequencing of DNA isolated from liver biopsy, collected after HSCT transplantation (Fig. S1G).

The MDS patient carried also a somatic Arg953* variant (c.2857C > T) in the paralog *STAG2* gene, annotated in InterVar, Varsome, and COSMIC as pathogenic in cancer (Fig. S2).

In order to set up an in vitro model to investigate the role in predisposition of *STAG1* germline mutations, two Lymphoblastoid Cell Lines (LCLs) were generated through the immortalization of PB B-lymphocytes from the Arg1167Gln mutated patient (L-STAG1) and from the Arg1187Gln mutated patient (M-STAG1). As a control, four different LCLs were generated from healthy donors.

First, we confirmed by PCR and Sanger sequencing that both L-STAG1 and M-STAG1 have maintained the genetic profile of interest (Figs. S1E–H; S2C), while the CTRs’ LCLs were wild type.

Second, we established the absence of other abnormalities in L-STAG1 and M-STAG1 LCLs, by karyotype and NGS custom panel analysis.

To evaluate the correlation between the different *STAG* variants and cancer, we analyzed the allele frequency (VAF) of the mutated positions across non-tumor and tumor cohorts. Known variants are combined and analyzed for the gene *STAG1*, transcript ID ENST00000383202. Minor allele frequencies of all coding germline variants present in *STAG1* in a global, healthy population, taken from the gnomAD database, are summed up codon-wise (Fig. S1B) and the VAF of *STAG1* p.1167 and p.1187 indicates that these mutations are rare in the general population (details in Supplementary Data).

An analogous model was applied to *STAG2* R953* somatic alteration, rarely germline mutated in the non-cancer population (gnomAD database, AF < 10^−5^) (Fig. S2D).

To investigate the functionality of *STAG1* variants on DNA stability, we evaluated the status of chromatin exchanges during the mitotic division.

**Fig. 1:**
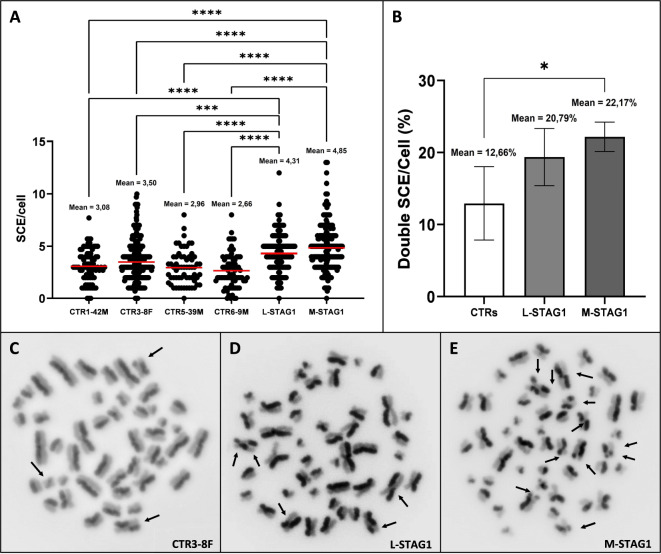
Sister chromatids exchange (SCE) incidence in LCL cells. **A** shows the higher number of SCE in L-STAG1 and M-STAG1 compared to control LCLs. **B** indicates the percentage of cells with double exchanges, which is significantly higher in M-STAG1. **C**–**E** show representative metaphases with single/double abnormal chromatid exchanges observed at fluorescence microscopy in CTR3-8F, L-STAG1, and M-STAG1, respectively. (Average of 88 metaphases for each line. Statistical analysis was performed by one-way ANOVA with Bonferroni’s multiple comparison correction. * <0.05; **<0.01 ***<0.001; ****<0.0001).

All LCLs were treated with phytohemagglutinin, to stimulate T lymphocyte growth (T0), incubated with *BrdU* (T24), which is incorporated only during the first mitotic division, and blocked in metaphase with Colchicine after the second generation (T72). Fluorescence microscopy after *Hoechst* staining showed that both M-STAG1 and L-STAG1 LCLs are characterized by a significantly higher number of abnormal chromatin exchanges. The average number of exchanges per nucleus is equal to 4.31 for L-STAG1 and 4.8 for M-STAG1, while the mean value for the four control LCLs is 3.05 (range 2.66 to 3.50; *p* < 0.0001) (Fig. 1A).

Furthermore, the population of cells that had one or more chromosomes with double exchanges is higher in both patients-derived LCLs: in L-STAG1 the percentage was 20.79%, compared to a mean of 12.66% for the control LCLs (*n* = 4; range 4.57 to 19.09%, *p* > 0.05), while in M-STAG1 the percentage was 22.17% (*p* = 0.0069) (Fig. 1B).

We also aimed to estimate the capability of LCLs to repair DNA after double-strand breaks (DSBs) induced by ionizing radiations. We evaluated the phosphorylation level of histone γH2AX, a common marker of DNA double-strand breaks damage [2, 11]. LCLs were seeded at different concentrations, to guarantee the exponential growth phase, they underwent X-ray irradiation at 3Gy and 6Gy and then were marked with Phospho-Histone H2AX antibody at different timepoints of incubation (T0, T24, T48 after irradiation).

**Fig. 2:**
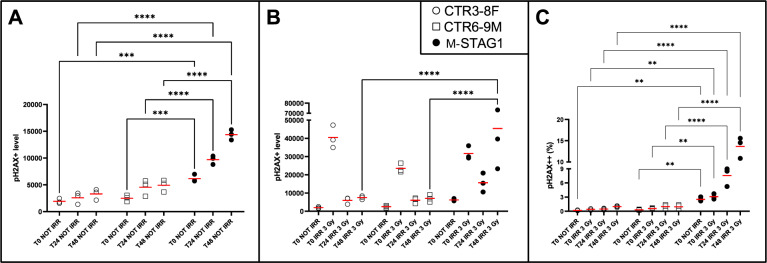
γH2AX phosphorylation status before and after X-ray irradiation. Cells were seeded at different, previously established, concentrations, in order to perform the experiments in an exponential growth phase (0.1 × 10^6^/ml for CTR3-8F, 0.22 × 10^6^/ml for CTR6-9M and 0.18 × 10^6^/ml for M-STAG1). **A** shows that γH2AX phosphorylation status of M-STAG1 is higher at basal conditions and increases during timepoints compared to control LCLs (T0: 2.8X, *p* < 0.001; T24: 2.7X, *p* < 0.0001; T48: 3.5X, *p* < 0.0001; MFI M-STAG1 over MFI control LCLs). γH2AX phosphorylation remains at higher levels also after irradiation [3Gy] (**B**, T24: 2.6X, ns; T48: 6.3X, *p* < 0.0001 [3Gy]; MFI M-STAG1 over MFI control LCLs, normalized on the percentage of γH2AX+ cells). The percentage of pH2AX++ subpopulation, recognized only for M-STAG1 either at basal level (10.5X, *p* < 0.01; percentage of pH2AX++ M-STAG1 cells over percentage of pH2AX++ control LCLs cells) or after irradiation (T0: 6.2X, *p* < 0.01; T24: 10.6X, *p* < 0.0001; T48: 14.1X, *p* < 0.0001; percentage of pH2AX++ cells M-STAG1 over percentage of pH2AX++ cells controls’ LCLs), shows the same trend (**C**). (*n* = 3 replicates. Statistical analysis performed by one-way Anova with Bonferroni’s multiple comparison correction. * <0.05; **<0.01 ***<0.001; ****<0.0001).

γH2AX phosphorylation status in M-STAG1 is significantly higher in basal conditions (Fig. 2A). The differential phosphorylation further increases after radiation at 3Gy or 6Gy. Fig. 2B shows a representative experiment at 3Gy, demonstrating a significantly lower capability of M*-*STAG1 to repair DNA after damage, compared to control LCLs.

Overall, control LCLs have a reduction of γH2AX phosphorylation at 48 h after irradiation (indicating a successful DNA repair), while M-STAG1 has an increased phosphorylation level, thus corresponding to a defective DNA repair capability.

In addition, a highly positive pH2AX subpopulation (namely pH2AX^++^) can be discriminated only in M-STAG1 even at basal conditions, and its level progressively increases at the timepoints after irradiation, thus confirming the previous result (Fig. 2C).

Similar findings have been obtained also after 6Gy irradiation (Fig. S7).

Therefore we identified two germline variants of the *STAG1* gene in two pediatric patients, affected by B-ALL and MDS, respectively. Those variants are located in a highly conserved region where multiple variants associated with solid tumors were mapped.

For the first time, we specifically explored the functional role of germline *STAG1* variants in oncogenesis, by evaluating how they can corrupt a pre-leukemic clone, making it genetically unstable and more prone to further somatic mutations.

We demonstrated that the *STAG1*-mutated LCLs have a higher number of both single and double chromatids exchanges compared to control LCLs. This is a common indicator of poor chromosomal strength and spontaneous chromosome instability, which is associated with failure of DNA repair and accumulation of DNA damage events. Similarly, higher SCE have been already found in other familial cancers, such as *BRCA1/2* mutated breast cancer [12].

Moreover, M-STAG1 cells displayed an increased DNA damage sensitivity, with a significantly lower DNA repair capability after X-ray irradiation. These results are consistent with studies by Bauerschmidt et al., who demonstrated that repair of radiation-induced DNA DSBs was reduced in *SMC1*- or *RAD21*-depleted cells [13].

The germline status of the identified variants in non-syndromic patients is compatible with their effects on DNA stability and DNA damage repair mechanisms, compatible with life but predisposing to oncogenesis.

Although the preliminary evidence on *STAG1* therapeutic potential [14], further biological studies are needed before considering the clinical relevance of *STAG1* germline variants and any therapeutic translation as a preemptive intervention. Taken together, our study provides strong evidence in support of the involvement of *STAG1* germline variants in predisposition to onco-hematological diseases in childhood.

If confirmed, cases carrying a *STAG1* germline variant would merit genetic counseling for the patient and its family, in order to make appropriate decisions for any therapeutic program (i.e., radiotherapy, selection of Hematopoietic Stem cell donor), as well as for any surveillance. It would be crucial to assess whether those variants have a *de novo* origin or silent carriers are present in the family.

In the future, we cannot exclude a similar scenario also involving other cohesin genes.

## Supplementary information

Saitta et al_supplementary_BCJ-0071RR

## References

[1] Solomon DA, Kim JS, Waldman T (2014). Cohesin gene mutations in tumorigenesis: from discovery to clinical significance. BMB Rep.

[2] Litwin I, Pilarczyk E, Wysocki R (2018). The emerging role of cohesin in the DNA damage response. Genes.

[3] Tothova Z, Valton AL, Gorelov RA, Vallurupalli M, Krill-Burger JM, Holmes A (2021). Cohesin mutations alter DNA damage repair and chromatin structure and create therapeutic vulnerabilities in MDS/AML. JCI Insight.

[4] Romero-Pérez L, Surdez D, Brunet E, Delattre O, Grünewald TGP (2019). STAG mutations in cancer. Trends Cancer.

[5] Piché J, Van Vliet PP, Pucéat M, Andelfinger G (2019). The expanding phenotypes of cohesinopathies: one ring to rule them all!. Cell Cycle.

[6] Jann J-C, Tothova Z (2021). Cohesin mutations in myeloid malignancies. Blood.

[7] Solomon DA, Kim JS, Bondaruk J, Shariat SF, Wang ZF, Elkahloun AG (2013). Frequent truncating mutations of STAG2 in bladder cancer. Nat Genet..

[8] Vial Y, Lachenaud J, Verloes A, Besnard M, Fenneteau O, Lainey E (2018). Down syndrome-like acute megakaryoblastic leukemia in a patient with Cornelia de Lange syndrome. Haematologica.

[9] Fazio G, Massa V, Grioni A, Bystry V, Rigamonti S, Saitta C (2019). First evidence of a paediatric patient with Cornelia de Lange syndrome with acute lymphoblastic leukaemia. J Clin Pathol.

[10] Hu Y, Caldwell KJ, Onciu M, Federico SM, Salek M, Lewis S (2022). CPX-351 induces remission in newly diagnosed pediatric secondary myeloid malignancies. Blood Adv.

[11] Sharma R, Lewis S, Wlodarski MW (2020). DNA repair syndromes and cancer: insights into genetics and phenotype patterns. Front Pediatr..

[12] De Pascalis I, Pilato B, Mazzotta A, Dell’Endice TS, Rubini V, Simone G (2015). Sister chromatid exchange: a possible approach to characterize familial breast cancer patients. Oncol Rep..

[13] Bauerschmidt C, Arrichiello C, Burdak-Rothkamm S, Woodcock M, Hill MA, Stevens DL (2010). Cohesin promotes the repair of ionizing radiation-induced DNA double-strand breaks in replicated chromatin. Nucleic Acids Res.

[14] Benedetti L, Cereda M, Monteverde LA, Desai N, Ciccarelli FD (2017). Synthetic lethal interaction between the tumour suppressor STAG2 and its paralog STAG1. Oncotarget..

