# Supplementary material for: Potential role of STAG1 mutations in genetic predisposition to childhood hematological malignancies
Source: Blood Cancer J. 2022 Jun 2;12(6):88. doi: 10.1038/s41408-022-00683-9 (PMC9163173; doi:10.1038/s41408-022-00683-9)
Supplement: Supplementary file 1 — Saitta et al_supplementary_BCJ-0071RR [file 41408_2022_683_MOESM1_ESM.docx]

# 1. SUPPLEMENTARY METHODS

**Ethic statement**

Samples were obtained from healthy donors and patients, with a written informed consent from patients or legal representatives. The study has been conducted in accordance with the ethical standards of the Declaration of Helsinki and to National and International guidelines. The study is approved by the institutional review board.

**DNA extraction**

Blood samples’ and LCLs’ DNA extractions were performed using the Wizard SV Genomic DNA Purification System (Promega Corp, Madison, WI, USA) according to protocol instructions.

DNA from the MDS patient’s liver biopsy was extracted from 5 slices (5 micron each) of a formalin-fixed, paraffin-embedded (FFPE) liver biopsy. DNA extraction was performed using Maxwell® RSC DNA FFPE Kit (Promega Corp., Madison, WI, USA) according to the manufacturer’s instructions. The sample was extracted with the automated DNA purification using the DNA IQ™ Casework Pro Kit for Maxwell® 16 (Promega Corporation, Madison, WI, USA).

**Next Generation Sequencing and bioinformatic data analysis**

A custom targeted Next Generation Sequencing (NGS) Nextera Flex DNA panel has been performed on bone marrow (BM) of hemato-oncological samples referred to our institution. Germline variants in Cohesin genes have been investigated both in disease and remission samples, defined by a minimal residual disease (MRD) value below 10^-4^. Sequencing has been performed by Nextseq550 (Illumina, San Diego, CA) in 2x150 paired end. FASTQ files are available in the ArrayExpress database ([www.ebi.ac.uk/arrayexpress](http://www.ebi.ac.uk/arrayexpress)), reference numbers E-MTAB-11757 and E-MTAB-11760.

Bioinformatic analysis was carried out by Sophia DDM software. Variants were filtered by variant fraction (VF)>5% and coverage at least 500X; Variant Allelic Fraction (VAF) in the population was set at 1%. We included pathogenic, potentially pathogenic and variants of unknown significance (VUS).

The most common databases of prediction were consulted for the interpretation of the pathogenicity, including: ClinVar, Clinical Genome, Varsome, InterVar, COSMIC. Benign/likely benign variants in all databases of prediction were excluded from the results (update April 2022).

***STAG1* variants and cancer**

The Arg1167Gln variant was identified in 3 cases out of 236700 individuals (GnomADv2.2.1 non-cancer; exome samples), while the Arg1187Gln variant was found in 1 case out of 147918 individuals (GnomADv3.1.2 non-cancer, genome samples). Somatic, coding variants reported for adult cancer patients derived from COSMIC, GRCh37 Release 91 (CosmicCodingMuts.normal.vcf.gz, n = 1,443,198 samples) are similarly combined for each codon along *STAG1*. Both collected datasets are smoothed using the LOWESS algorithm (fraction: 0.06, iteration: 1) prior to plotting.

**RT-PCR for variants validation**

RT-PCR was performed using primers in the *STAG1* and *STAG2* mutated exons (Supplementary Table S2).

All RT-PCR reactions were performed at the following conditions: denaturation for 2’ at 94°C, then thirty-five cycles of amplification (30 s at 94°C, 30 s at 60°C, 60 s at 72°C), using the Platinum SuperFi II DNA Polymerase–High-Fidelity PCR Enzyme (Life Technologies, Thermo Fisher, Carlsbad, California, United States).

**Lymphoblastoid Cell Lines**

Lymphoblastoid cell lines (LCLs) were derived from *in vitro* transformation and immortalization of B lymphocytes in fresh peripheral blood (PB) by Epstein Barr virus (EBV) (BioBank Service, Gaslini Hospital, Genova, Italy). All cells were tested for mycoplasma.

Cells were grown in T25 flasks in RPMI medium with 10% FBS, 1% Pen-Strep and 1% L-glutamine, in standard incubation conditions (37 °C, 5% CO_2_).

**Phenotype characterization**

A flow cytometry antibody panel was developed to characterize LCL B-cell phenotype, including specific antibodies for B-cells, T-cells and myeloid cells markers, such as CD19 (FITC, #11-0199-42, eBioscience™), CD45 (PO, #MHCD4530, Invitrogen™, Waltham, Massachusetts, US), CD3 (Alexa700, #557943, Becton Dickinson™), CD13 (PE, #347406, Becton Dickinson™) and CD33 (PeCy7, #333952, Becton Dickinson™) in addition to the stemness marker CD34 (PerCPCy5.5, #347222, Becton Dickinson™). After 30’ incubation (RT, in the dark), cells were washed and resuspended in 200 μl of PBS and analyzed with BD LSRFortessa™ X-20 Flow Cytometer, BD FACSDiva™ software (BD Biosciences) and FlowJo software (Tree Star, Inc. Ashland, OR, USA).

**Cell Growth**

To evaluate the growth rate, LCLs were seeded at different concentrations according to their previously established growth characteristics: 0.1x10^6^/ml for CTR3-8F, 0.22x10^6^/ml for CTR6-9M and 0,18x10^6^/ml for M-STAG1 (MW6 plates). Cells were collected after 72, 96 and 120 hours. Live cells were counted by Trypan Blue exclusion both through Countess Automated Cell Counter (Thermo Fisher, Carlsbad, California, United States) and Burker’ counting chamber at optical microscope, in parallel, considering the mean of the counts. Detailed data in supplementary Figure S4.

**Cell Cycle Assay**

Basal and irradiated cells were collected in polypropylene tubes at 2x10^6^/ml. After a centrifugation (1800 rpm, 5’) the pellet was resuspended on ice in 1 mL of GM saline buffer (Glucose 1.1 g/l, NaCl 8 g/l, KCl 0.4 g/l, Na_2_HPO_4_.2H_2_O 0.2 g/l, KH_2_PO_4_ 0.15 g/l, EDTA 0.5M 0.2 g/l). 1.3 mL of 96% Ethanol were then added under stirring for each sample. The fixed samples, stored at + 4 °C or at -20 °C, were centrifuged (1200 rpm, 10’) and then washed with 1 mL PBS. They were subsequently incubated at 4°C overnight in the dark with 1 mL solution of Propidium Iodide (2.5 µg/mL) and 12.5 µl of RNase (1 mg/mL). Flow cytometry analysis was executed using the BD LSRFortessa™ X-20 instrument and BD FACSDiva™ software. Cell cycle analysis was performed on at least 20000 cells for each. Cell cycle phase distribution was calculated as percentages by a Gaussian-modified method [1]. Detailed data in supplementary Figure S5.

**Sister Chromatids Exchange Assay**

One ml of LCLs cell culture suspension (1x10^6^ cells in 5 ml RPMI 10% FBS at conc. 0.3x10^6^/ml) was added to 7 ml of medium and 250 µl of Phytohemagglutinin to stimulate T lymphocytes growth. After an incubation at 37°C for 24h, 80 µl of a 1 µg/µl BrdU stock solution was added, and samples were incubated at 37°C for 48h. In this condition, cells grow and replicate, and new synthesized DNA will not be marked with BrdU, thus allowing the visualization of chromosomal exchanges. Colchicine has been added to block the mitotic spindle during the metaphase. Samples were incubated for 1.5 h and then transferred into 15 mL Falcon tubes and centrifuged at 1800 rpm (10’). Cells were resuspended in 7 mL of hypotonic solution (KCl 0.08 M) and incubated at 37°C for 15’. 1 mL of fixative solution (methyl alcohol and acetic acid in ratio 3:1) was added to the samples which were centrifuged at 1800 rpm (10’) and then resuspended in 7 mL of fixative. The pellet was resuspended in 2 mL of fixative solution and smeared on a cold glass slide. Slides were stained with 10 µl of Hoechst (1:5000) each and incubated for 20’ to visualize the frequency of SCE through a fluorescence microscope.

**X-ray irradiation**

LCLs underwent a cycle of X-ray irradiation, either at 3 Gy (190 V, 12 A, 5.5’) and 6 Gy (190 V, 12 A, 11’), on the RADGIL instrument (Gilardoni SpA, Mandello del Lario, Italy).

**pH2AX level evaluation**

To investigate the capability of LCLs to repair DNA after double-strand breaks (DSBs) induced by an ionizing radiation, we evaluated the phosphorylation level of γH2AX, a DSB marker, by FACS analysis.

Cells were seeded in MW6 at the same concentration and conditions used for the growth curves, in order to perform the experiments in an exponentially growing phase.

Approximately 1x10^6^ of basal or irradiated cells per sample were collected in FACS tubes and centrifuged at 1200 rpm (5’). Cells were resuspended in 1 mL of PBS and 2 mL of fixative solution (4.5% PFA/PBS, 3% final concentration). Samples were incubated for 10 min (RT). After a centrifugation (1200 rpm, 5’) the pellet was resuspended in 3 mL of cold Ethanol 70% and vortexed briefly.

To remove ethanol, the cell pellet was washed 3 times in 3 mL of washing solution (0.5% BSA/PBS), resuspended with Phospho-Histone H2AX antibody (Alexa Fluor® 488 Conjugate - BD #9719) and incubated for 1h at RT. Samples were centrifuged at 1800 rpm for 5 minutes and resuspended in 200 µl of PBS.

Flow cytometry analysis was performed using the BD LSRFortessa ™ X-20 instrument, BD FACSDiva software and FCS Express Flow Cytometry from De Novo Software.

**Statistical analysis**

Results are expressed as mean values of technical replicates. All the experiments were performed at minimum in triplicate, evaluating standard deviation. This criterion was applied for both control and mutated samples.

Statistical analysis was performed by Graphpad Prism software ver.9.2.0 one-way ANOVA test with Bonferroni's multiple comparisons is shown as *p<0.05, **p<0.01, ***p<0.001.

# 2. SUPPLEMENTARY RESULTS

**LCL phenotype characterization**

In order to assess that LCLs have maintained the B-lineage profile after EBV immortalization, a flow cytometry antibody panel was developed to characterize their phenotype.

We evaluated specific markers of hematopoietic subpopulations, including common lymphocyte markers (the pan-leukocyte hCD45 and hCD19 for B-cells and hCD3 for T-cells), myeloid markers (hCD13 and hCD33) and a stemness marker (hCD34). The results show a marked positivity against hCD45 and hCD19 antibodies, confirming the immortalization of the B-cell subpopulation. The results are comparable in all LCLs tested, derived both from healthy donors and mutated patients (Figure S3).

**M-STAG1 and control LCLs growth is affected by X-ray irradiation.**

To evaluate the different growth rates of LCLs, firstly, we tested different seeding cells for each line, in order to identify the best individual conditions that guarantee for each the exponential growing phase. On the bases of the results, we set up the experimental conditions to compare M-STAG1 with mean of control LCLs and we demonstrated that in basal condition the growth ratio for each timepoint over the previous one is comparable between the cell lines. (Ratio T24/T0 1.41 for M-STAG1 over 1.50 for controls’ mean, p> 0.05 n.s.; T48/T0: 1.29 for STAG1_LCL over 1.14 for controls’ mean, p>0.05 n.s. One-sample T-Test). After X-ray irradiation, both M-STAG1 and control LCLs are characterized by a remarkable reduction in terms of growth capability in response to the damage stimulus, but the trend remains similar as shown in Figure S4.

**M-STAG1 and control LCLs cell cycle is affected by X-ray irradiation -G2M block**

To assess the different distribution in cell cycle phases, we evaluated the percentage of cells in each cell cycle phase (G0/G1; S; G2/M) for each LCLs.

Basal and irradiated cells (2x10^6^/ml for each condition) were resuspended in GM saline buffer (Glucose 1.1 g/l, NaCl 8 g/l, KCl 0.4 g/l, Na_2_HPO_4_.2H_2_O 0.2 g/l, KH_2_PO_4_ 0.15 g/l, EDTA 0.5M 0.2 g/l), fixed with 96% Ethanol and incubated with propidium iodide (2.5 µg/mL) and 12.5 µl of RNase (1 mg/mL). Flow cytometry analysis was executed using the BD LSRFortessa™ X-20 instrument and BD FACSDiva™ software. Cell cycle analysis was performed on at least 20000 cells for each. Cell cycle phase distribution was calculated as percentages by a Gaussian-modified method [1].

We didn’t appreciate any significant difference between CTRs and M-STAG1 in basal condition. This trend is comparable with the growth rate observed in cell growth curves.

Only when referring to timepoints 24h and 48h, we found a slight difference in G0/G1 and S phases in M-STAG1 compared to CTRs, where mutated cells seem to have a higher percentage of cells in S phase. This trend does not persist in the ulterior timepoints.

After X-ray irradiation, cell cycle perturbations are comparable across LCLs lines. As shown in figure S5, a G2M block induced by 3 Gy and 6 Gy was detected both in control LCLs and M-STAG1.

**Defective capability of M-STAG1 to repair DNA after an ionizing radiation at 6Gy**

The γH2AX phosphorylation status of M-STAG1 remains at higher levels than control LCLs also after a higher ionizing radiation [6Gy] (T24: 1.8X, ns; T48: 7.3X, p<0.0001 [6Gy]; MFI M-STAG1 over MFI controls’ LCLs normalized on the percentage of γH2AX^+^ cells) (Figure S7, panel A). These data confirmed a significantly lower capability of M-STAG1 to repair after a DNA damage, compared to controls’ LCLs. Moreover, they revealed that more intense X-ray dosage causes a higher DNA damage, thus mutated cells are more impaired in repairing.

The percentage of pH2AX^++^ subpopulation in M-STAG1 is characterized by the same trend. (T24: 8.8X, p <0.0001; T48: 15.2X, p <0.0001; percentage of pH2AX^++^ cells M-STAG1 over percentage of pH2AX^++^ cells controls’ LCLs) (Figure S7, panel B).

**Figure Legend**

Figure S1. Validation and analysis of *STAG1* variants.

In panel A, the germline ALL-mutation (Arg1167Gln/R1167Q; bordeaux label), the germline MDS-mutation (Arg1187Gln/R1187Q; red label) and the other somatic variants previously described (blue label) on *STAG1*. In panel B distribution of variants frequencies along *STAG1*, based on two databases: the top shows the adjusted AF (%) of variants in the gnomAD non-cancer database, while the bottom shows the adjusted frequency of variants in the COSMIC (somatic cancer mutations) database. In panels C-D-E, chromatograms of bone marrow ALL patient at diagnosis, bone marrow sample at remission phase and L-STAG1, respectively. In panels F-G-H, chromatograms of bone marrow MDS patient at diagnosis, sample from liver biopsy and M-STAG1, respectively.


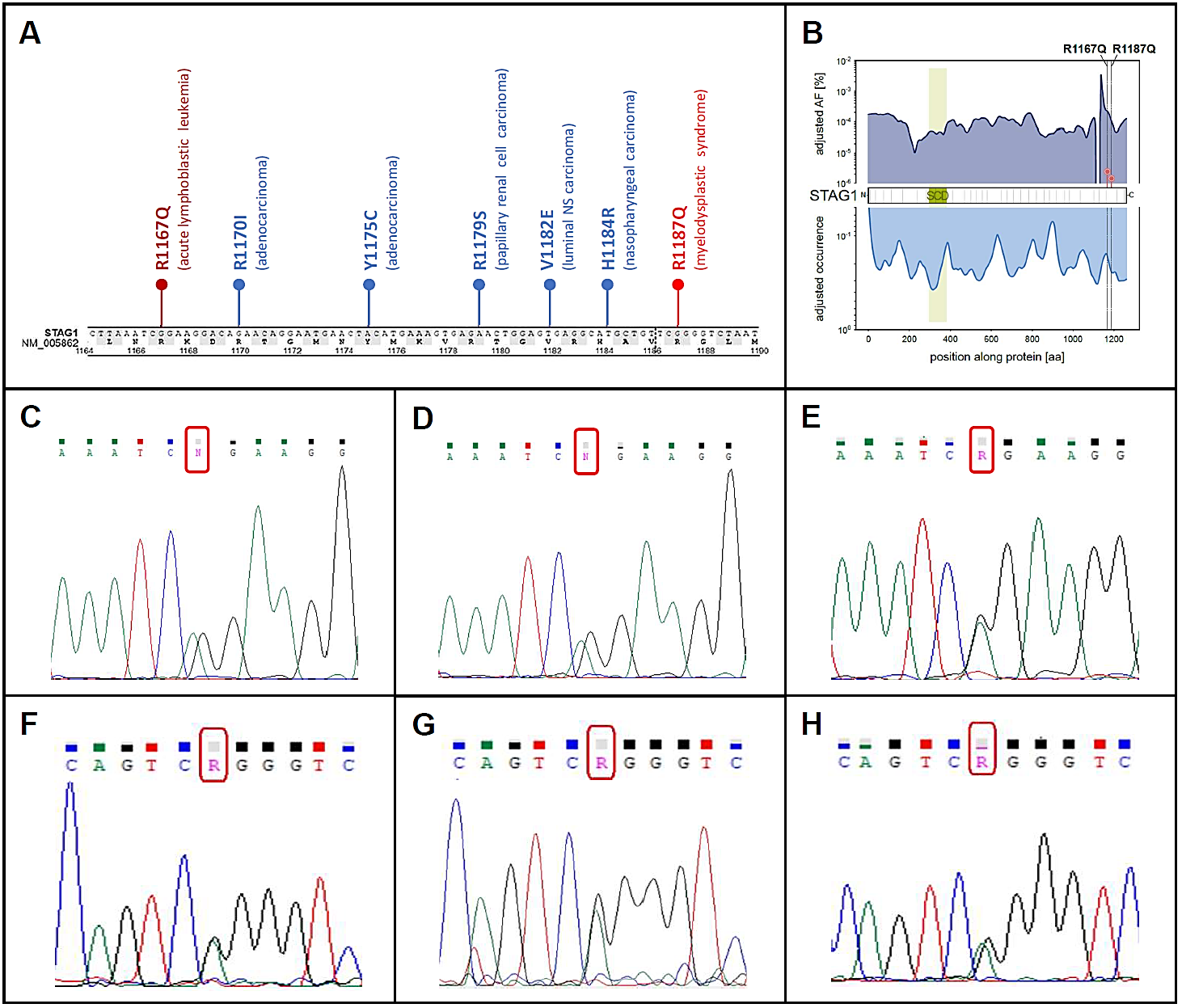


Figure S2. Validation and analysis of *STAG2* variant.

In panel A, somatic *STAG2* variant (Arg953*/R953*; red label) of MDS patient. In panels B-C, chromatograms of bone marrow MDS patient at diagnosis and M-STAG1, respectively. In panel D distribution of variants frequencies along *STAG1*, based on two databases: the top shows the adjusted VAF (%) of variants in the gnomAD non-cancer database, while the bottom shows the adjusted frequency of variants in the COSMIC (somatic cancer mutations) database.


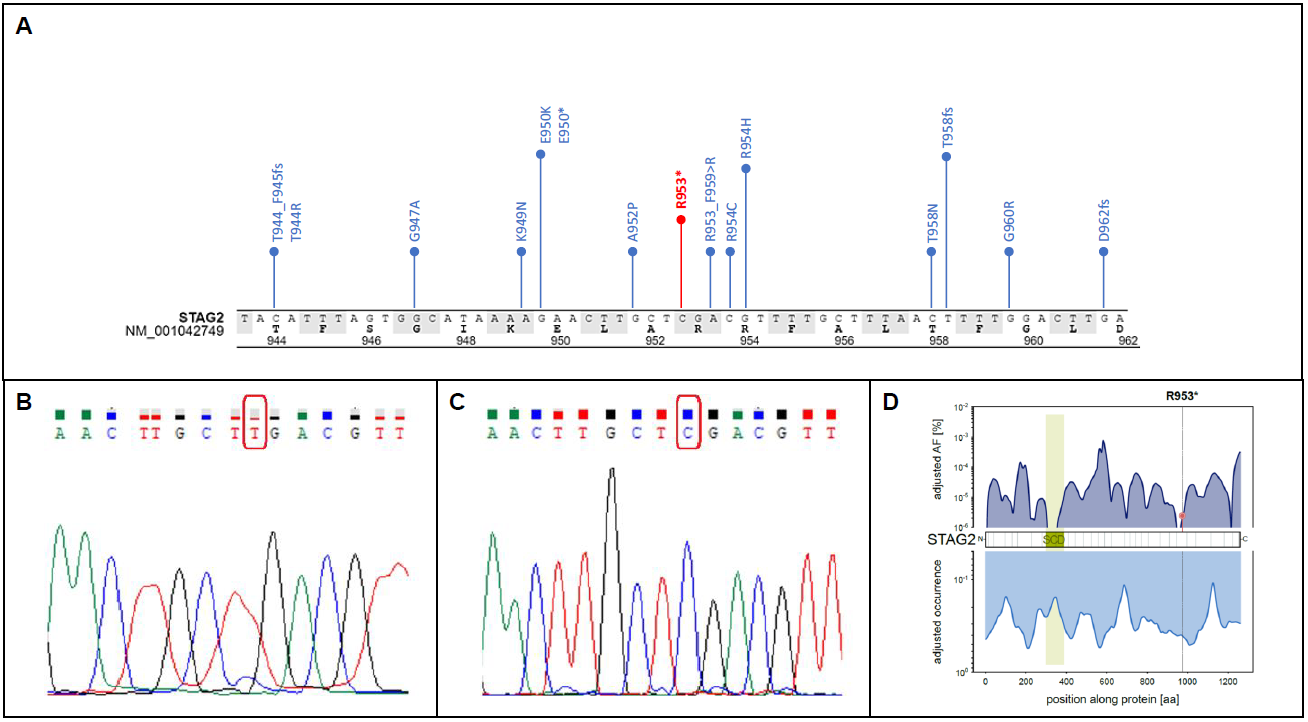


Figure S3. Phenotype characterization on LCLs.

In panels A-D dotplots of hCD19^+^/hCD45^+^ cells in CTR3-8F_LCL, CTR6-9ML, L-STAG1 and M-STAG1, respectively. In panels E-F are represented overlay histograms of hCD45^+^ and hCD19^+^ cells in each LCL, compared to unstained LCLs.


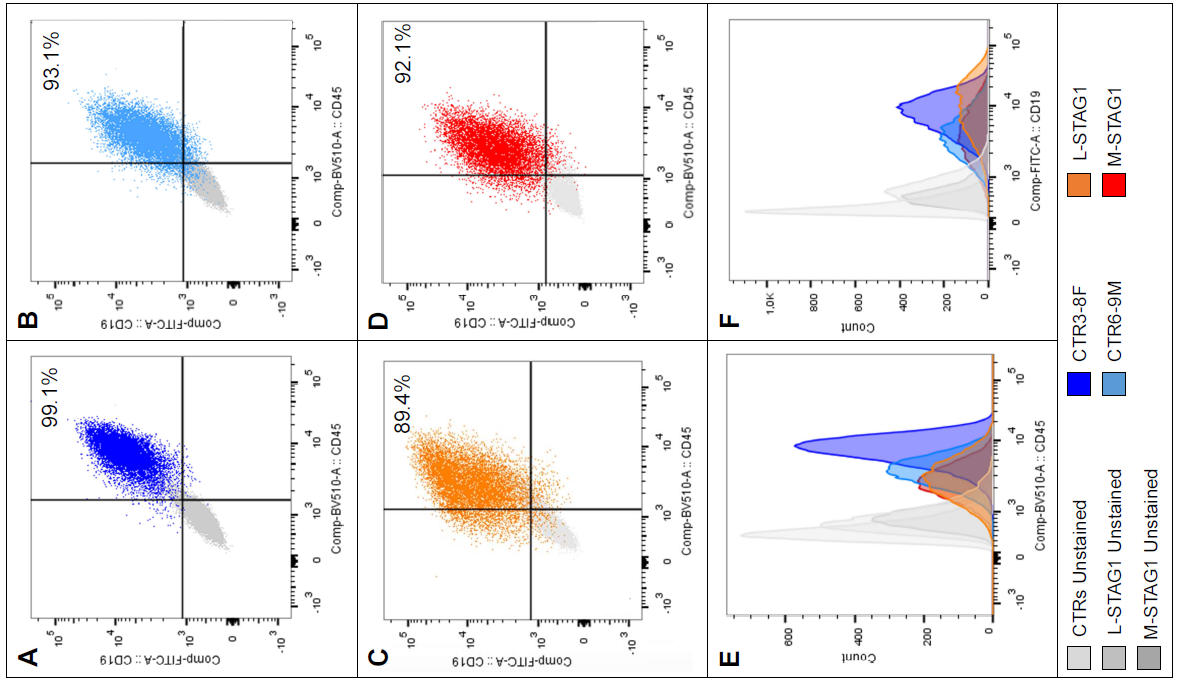


Figure S4. Growth curves of LCLs, before and after X-ray irradiation.

The reduction of cells growth rate is comparable between M-STAG1 and the mean of the two control LCLs, either after a 3Gy irradiation (A-B-C) or after a 6Gy irradiation (D-E-F).(Ratio T24/T0 0.99 for M-STAG1 over 0.96 for controls’ mean, p> 0.05 n.s.; T48/T0: 0.92 for M-STAG1 over 0.95 for controls’ mean, p>0.05 n.s. [3Gy]; Ratio T24/T0 0.90 for M-STAG1 over 1.01 for controls’ mean, p> 0.05 n.s.; T48/T0: 0.87 for M-STAG1 over 0.82 for controls’ mean, p>0.05 n.s. [6Gy]. (Statistical analysis performed by One-sample T-Test. * <0,05; **<0,01 ***<0,001; ****<0,0001)


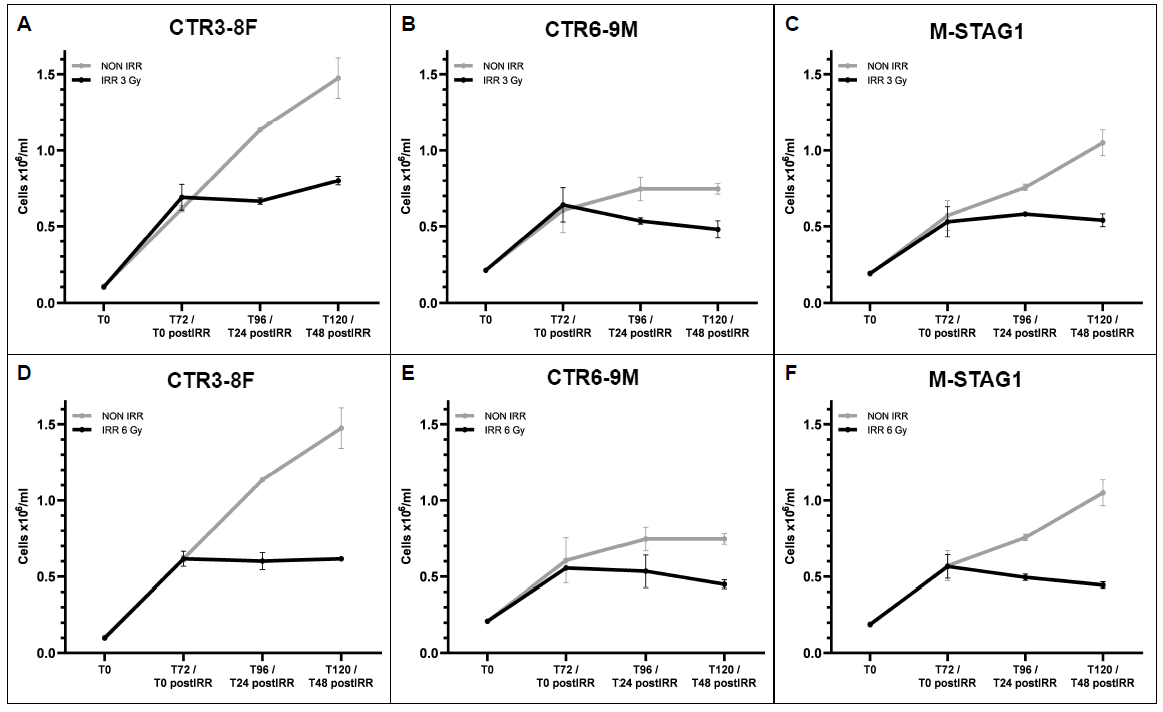


Figure S5. Effect of X-ray irradiation on the cell cycle in LCLs.

Cell cycle phase perturbations induced by [3Gy] [6Gy] irradiation on CTR3-8F_LCL (A), CTR6-9M_LCL (B) and M-STAG1 (C) after 24, 48 and 72 h after damage stimulus.


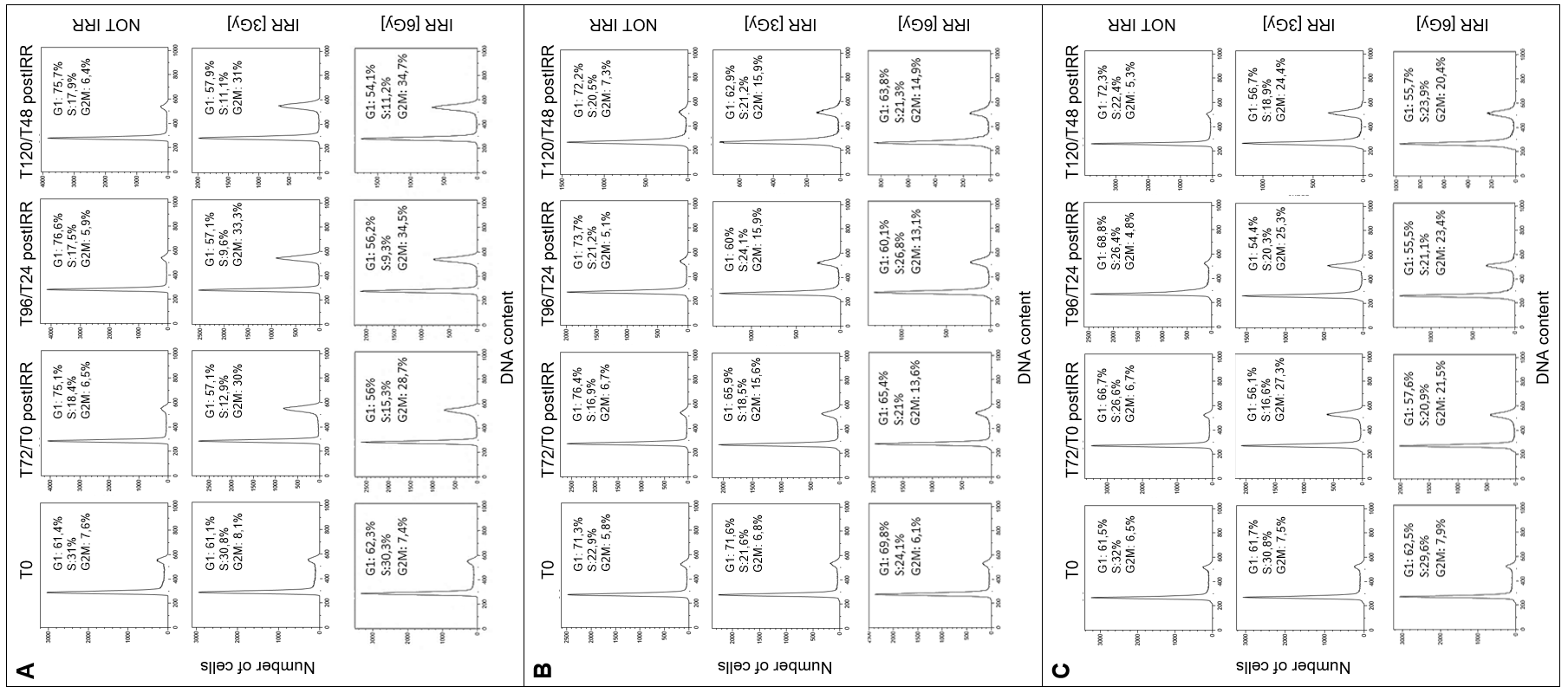


Figure S6. Representative dot plots of γH2AX phosphorylation status before and after X-ray irradiation [3Gy] – Timepoints 0h, 24h and 48h.

CTR3-8F_LCL and CTR6-9M_LCL in the first two columns and M-STAG1 in the third one. In the panels A-B-C not irradiated cells; in the panels D-E-F phosphorylation status at T0 after irradiation; in the panels G-H-I and J-K-L reduction of pH2AX^+^ cells at T24 and T48 can be appreciated, expression of different capability to repair after a DNA damage between control LCLs and M-STAG1.


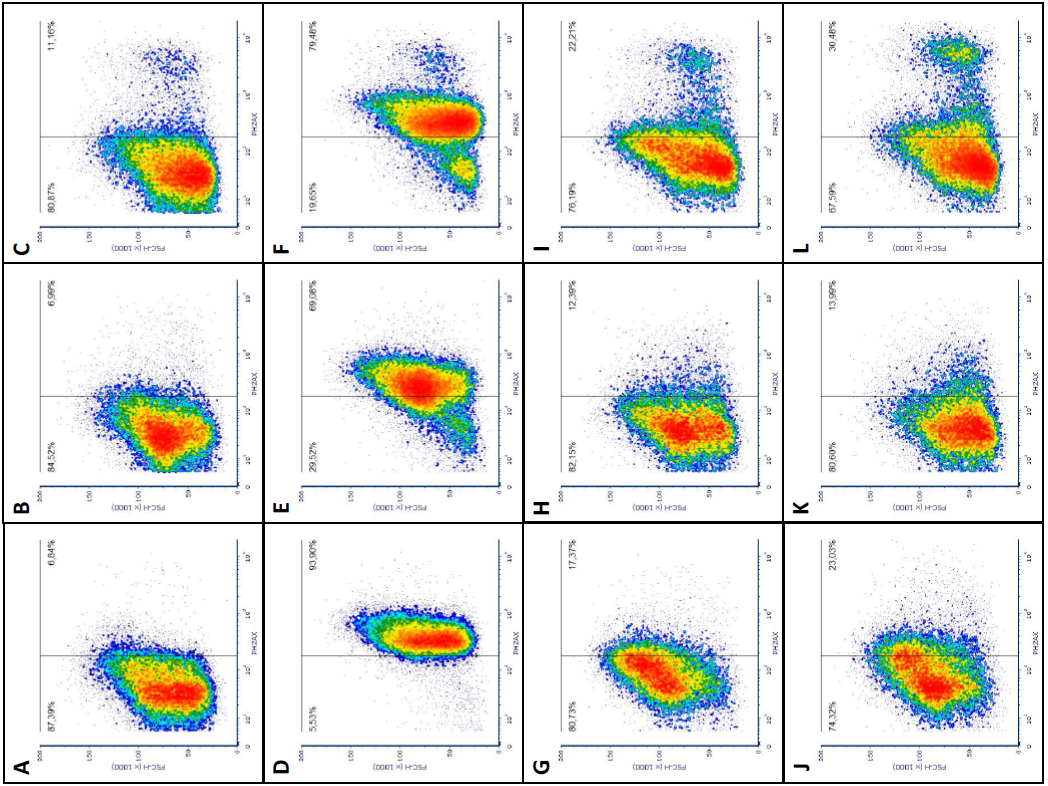


Figure S7. γH2AX phosphorylation status before and after an X-ray irradiation [6Gy].


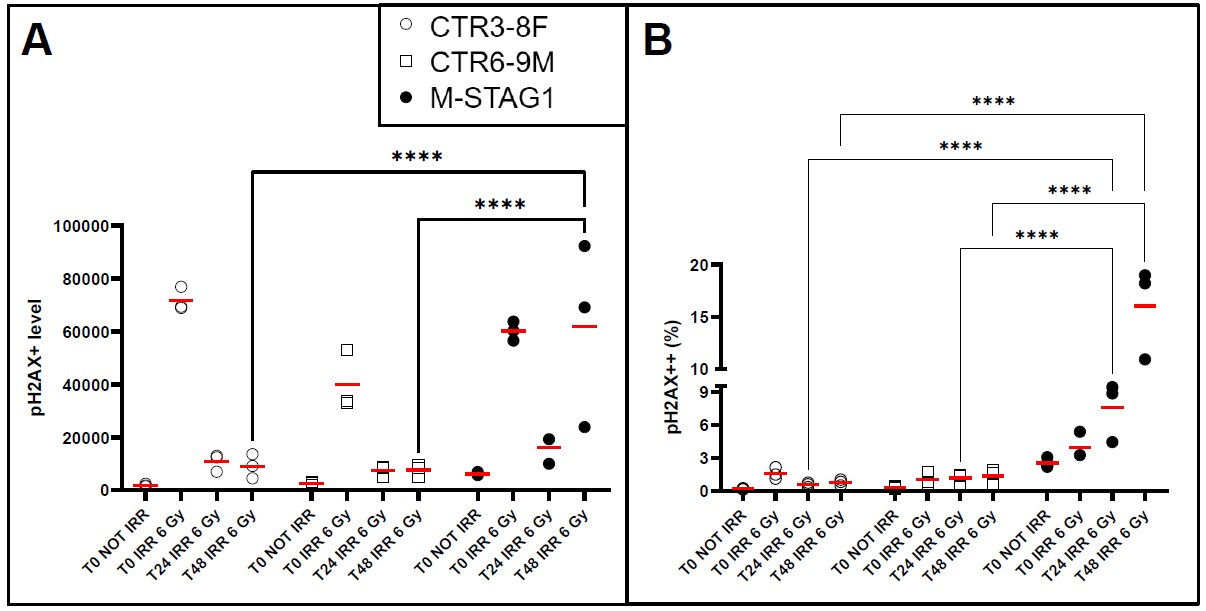


After a higher X-ray irradiation [6Gy], γH2AX phosphorylation status remains at higher levels in M-STAG1 compared to controls’ LCLs (panel A). The percentage of pH2AX^++^ subpopulation (panel B) shows comparable results. (n=3 replicates. Statistical analysis performed by One-way Bonferroni’s multiple comparison correction. * <0,05; **<0,01 ***<0,001; ****<0,0001)

Table S1. Categories of the 39 cancer genes analyzed for mutations.

Classification of genes included in custom Next Generation Sequencing panel in different classes according to their biological functions.


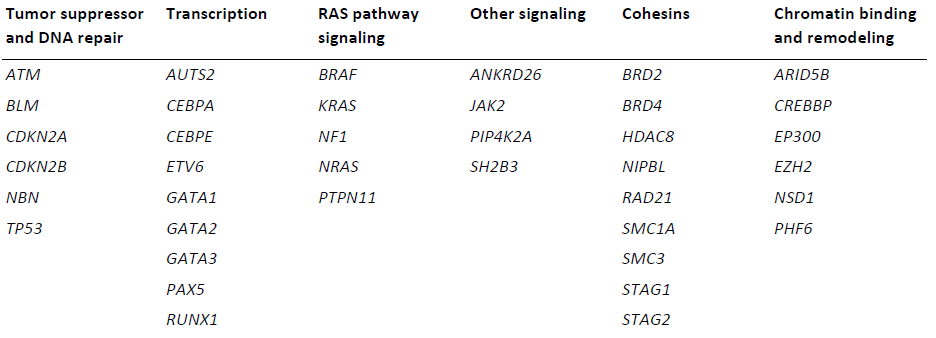


Table S2. *STAG1* and *STAG2* mutations validation RT-PCR primers.


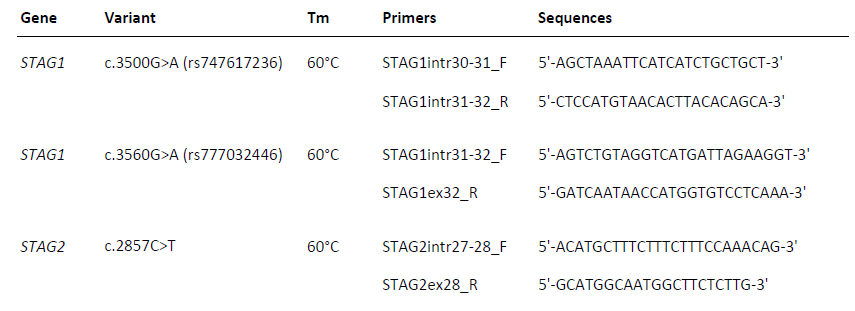


SUPPLEMENTARY REFERENCE

1. Ubezio P. Microcomputer experience in analysis of flow cytometric DNA distributions. Comput. Programs Biomed. 1985*;* 19:159–166.
